# Prognostic significance of left atrial reservoir strain during exercise in patients with heart failure

**DOI:** 10.1016/j.ijcha.2026.102001

**Published:** 2026-08-24

**Authors:** Yu Yoshida, Yoichi Takaya, Rie Nakayama, Rika Takemoto, Eriko Kusunoki, Norihisa Toh, Toru Miyoshi, Shinsuke Yuasa

**Affiliations:** a Department of Cardiovascular Medicine, Okayama University Graduate School of Medicine, Dentistry and Pharmaceutical Sciences, Okayama, Japan; b Division of Medical Support, Okayama University Hospital, Okayama, Japan

**Keywords:** Exercise stress echocardiography, Heart failure, Left atrial reservoir strain, Prognosis

## Abstract

**Background:**

This study aimed to evaluate the association of peak exercise left atrial (LA) reservoir strain with clinical outcomes in patients with heart failure (HF), and the prognostic value across HF phenotypes.

**Methods:**

We enrolled 150 patients with HF (age 67 ± 15 years, 61% males) who underwent exercise stress echocardiography. LA reservoir strain at rest and peak workload were evaluated using the speckle-tracking echocardiography. The endpoint was a composite of cardiovascular death or hospitalization for HF.

**Results:**

During a median follow-up of 1.1 years (interquartile range 0.7–2.4 years), 49 patients experienced events. Patients were categorized into two groups on the basis of the median value of LA reservoir strain at peak workload of 16.7%. Kaplan–Meier curves showed that patients with low LA reservoir strain at peak workload had a higher event rate than those with high LA reservoir strain (log-rank test, *P* < 0.001). LA reservoir strain at peak workload was independently associated with events (hazard ratio: 5.57, 95% confidence intervals: 2.74–11.3, *P* < 0.001), and provided the incremental prognostic value over clinical factors. When analyzed according to HF phenotype, LA reservoir strain at peak workload was significantly associated with events in both patients with HFpEF and those with HFrEF, with a significant interaction between HF phenotype and LA reservoir strain at peak workload.

**Conclusion:**

LA reservoir strain at peak workload has prognostic value for adverse outcomes in both HFpEF and HFrEF.

## 1 Introduction

The left atrium (LA) plays a dual role as a low-pressure reservoir and an active contractile chamber. The structural and functional remodeling of the LA often precedes left ventricular (LV) dysfunction, reflecting early pathophysiological changes, such as elevated LV filling pressures [1], [2], [3]. LV volume or pressure overload induces LA enlargement and decreased LA compliance [4]. Two-dimensional speckle-tracking echocardiography enables quantification of phasic LA functions—reservoir, conduit, and booster pump—providing a non-invasive insight into LA mechanics [5]. Especially, LA reservoir strain correlates with elevated LV filling pressures and serves as a prognostic marker linked to symptoms, functional status, and adverse outcomes [6], [7], [8].

Clinical symptoms, such as dyspnea and fatigue, are more pronounced during exercise in patients with heart failure (HF), reflecting the inability of the cardiovascular system to adequately augment cardiac output in response to physiological demand. Accordingly, exercise stress echocardiography is increasingly used to unmask latent hemodynamic abnormalities [9], [10], [11]. Current recommendations emphasize the role of exercise stress echocardiography in the evaluation of unexplained exertional dyspnea when resting echocardiographic findings are inconclusive [12], [13]. While LV diastolic dysfunction has been considered the main driver of exercise intolerance in HF, recent studies highlight the pivotal role of LA reservoir function in modulating LV filling pressures [14], [15]. LA reservoir function during exercise, as reflected by peak exercise LA reservoir strain, may provide incremental prognostic value in patients with HF. Additionally, HF is a heterogeneous syndrome and is commonly classified into distinct phenotypes based on LV function, namely HF with preserved ejection fraction (HFpEF) and HF with reduced ejection fraction (HFrEF), which differ in underlying pathophysiology and hemodynamic characteristics. The prognostic impact of peak exercise LA reservoir strain may differ between HF phenotypes. However, limited information is available about the utility of peak exercise LA reservoir strain for predicting clinical outcomes, including across HF phenotypes.

We therefore hypothesized that LA reservoir strain during exercise is associated with clinical outcomes in both HFpEF and HFrEF, but that the strength of this association may differ between HF phenotypes. Accordingly, the aim of this study was to evaluate the association between LA reservoir strain at peak workload and clinical outcomes in patients with HF, and the prognostic value across HF phenotypes.

## 2 Methods

### 2.1 Study population

**Fig. 1:**
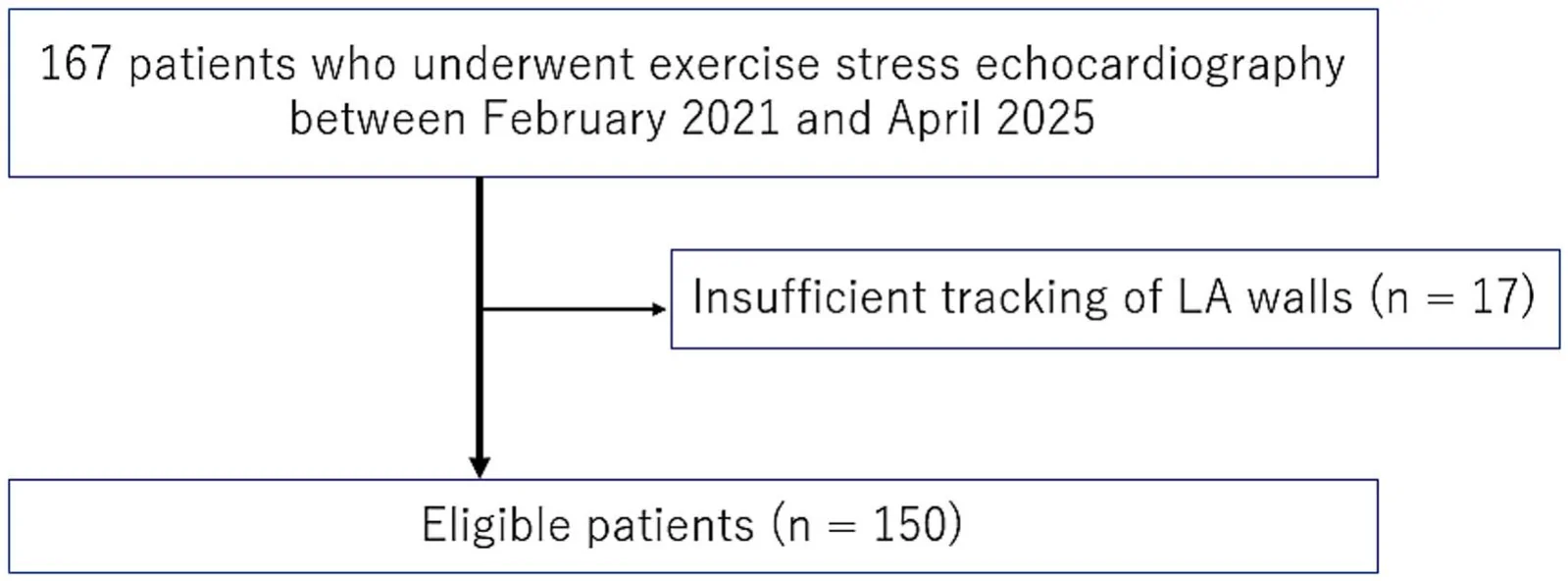
Study flow diagram. Flowchart illustrating patient inclusion and exclusion.

We prospectively enrolled 167 consecutive patients with HF who underwent exercise stress echocardiography between February 2021 and April 2025 at Okayama University Hospital. Exercise stress echocardiography was performed as clinical evaluation of HF severity at the discretion of the attending physician. Seventeen patients with poor image quality for insufficient tracking of the LA walls were excluded. Finally, 150 patients were enrolled in the study (Fig. 1). All patients had clinical symptoms or signs of HF, according to the 2021 European Society of Cardiology guidelines [16]. Clinical characteristics, comorbidities, medical therapy, laboratory measurements such as hemoglobin, albumin, estimated glomerular filtration rate, and plasma B-type natriuretic peptide or N-terminal pro-B-type natriuretic peptide levels, and echocardiographic findings were obtained.

This study was conducted following the principles of the Declaration of Helsinki and reviewed and approved by the ethics committee of Okayama University Graduate School of Medicine, Dentistry and Pharmaceutical Sciences. Informed consent was obtained before entering this study.

### 2.2 Echocardiographic parameters

Two-dimensional and Doppler transthoracic echocardiography (TTE) were performed by experienced sonographers using a commercially available ultrasound system (Aplio i900, Canon Medical Systems, Ohtawara, Japan; EPIQ, Philips Medical Systems, Andover, MA, USA; Vivid E95, GE Healthcare, Horten, Norway). TTE measurements were performed according to the guidelines [17]. LV volumes and LV ejection fraction were assessed from apical four-chamber views. Early diastolic transmitral inflow velocity (E) and early diastolic mitral annular velocity at septal wall (e’) were measured, and E/e’ ratio was calculated. Stroke volume was derived from LV outflow tract and pulse-wave Doppler profile, and cardiac output was calculated by multiplying heart rate. Tricuspid regurgitation (TR) pressure gradient was measured from the peak velocity.

### 2.3 LA function assessment

**Fig. 2:**
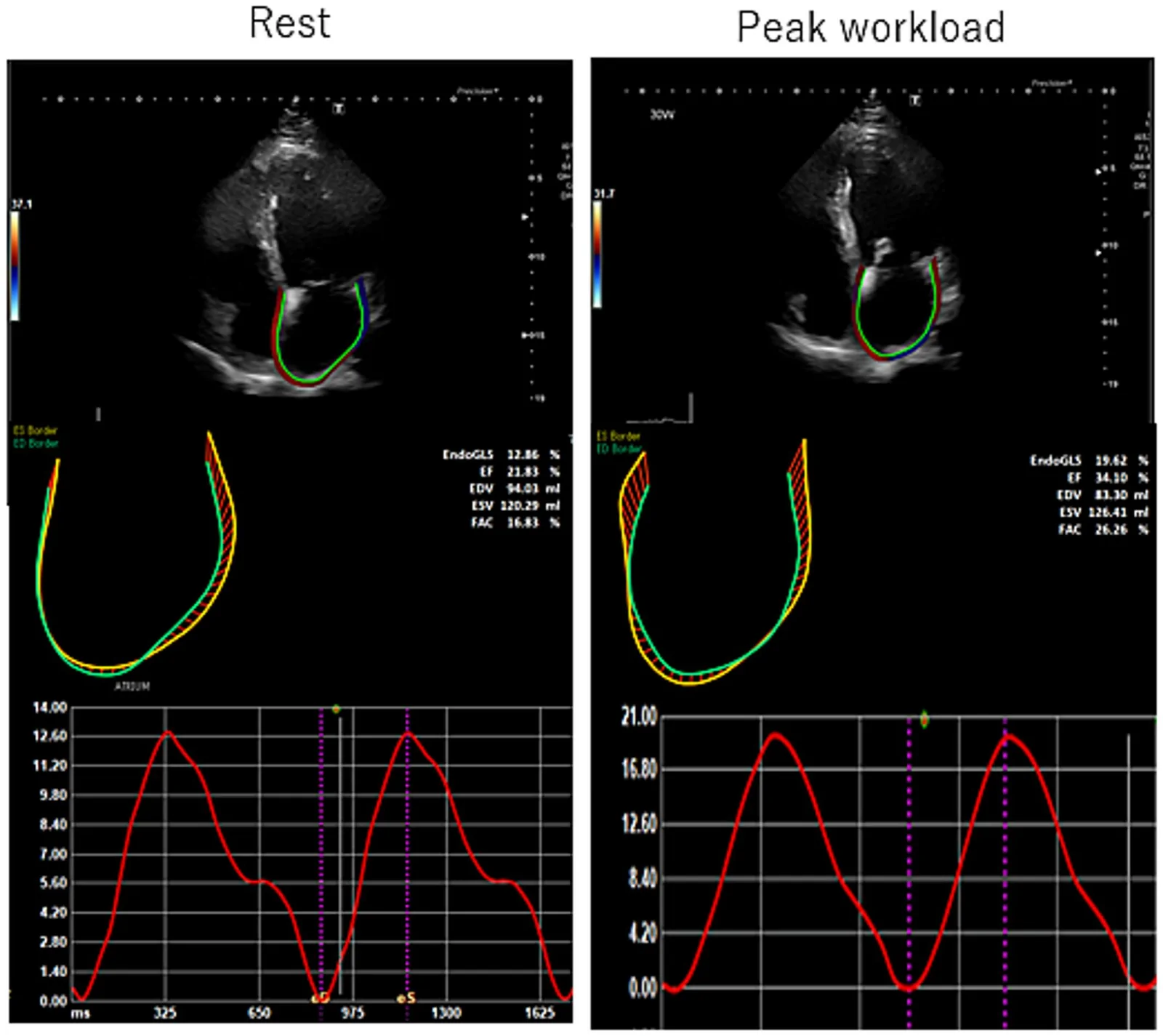
Representative images of LA reservoir strain at rest and peak workload. The speckle-tracking echocardiographic analysis of LA reservoir strain obtained from the apical four-chamber view. The left panel shows LA reservoir strain at rest of 12.9%. The right panel shows LA reservoir strain at peak workload of 19.6%. LA, left atrial.

LA function was assessed using vendor-independent software (TOMTEC Imaging System, Unterschleissheim, Germany) (Fig. 2). All echocardiographic images were sent in DICOM format to the core laboratory. The speckle-tracking echocardiography was analyzed offline from apical four-chamber view images. Three reference points were placed at the mitral annulus and the roof of the LA. The software automatically determined the LA endocardial border and performed speckle-tracking analysis through one complete cardiac cycle. When an inaccurate LA endocardial border was detected, manual adjustment was performed to ensure optimal tracking. The speckle-tracking echocardiography provided values for average LA reservoir strain, LA volume, LA fractional area change and LA ejection fraction. LA volume index was calculated by body surface area. The speckle-tracking echocardiography analyses were performed by experienced and independent cardiologists who were blinded to the patients' clinical information.

### 2.4 Exercise stress echocardiography

All patients underwent exercise stress echocardiography using a symptom-limited bicycle ergometer in a supine, slightly left lateral decubitus position on a tilting table. The initial workload (10 W or 20 W) was selected by the attending physician according to each patient's physical condition, frailty, and estimated exercise capacity. The exercise intensity was increased stepwise by 10 or 20 W every 2 min until exhaustion while maintaining a pedaling speed of 60 rpm. Electrocardiogram and oxygen saturation were monitored continuously. Blood pressure and heart rate were recorded every 2 min. Exercise was interrupted in the event of chest pain, ST-segment shift, excessive blood pressure increase (systolic blood pressure ≥ 240 mmHg, diastolic blood pressure ≥ 120 mmHg), limiting dyspnea, or significant arrhythmias. Echocardiographic images were obtained at rest and during exercise. E, e’, E/e’, TR pressure gradient, stroke volume, cardiac output, and LA reservoir strain were measured at rest and peak workload.

### 2.5 Clinical outcomes

The endpoint was a composite of cardiovascular death or hospitalization for HF. The definition of cardiovascular death comprised death resulting from HF, myocardial infarction, or other cardiovascular causes, as well as sudden cardiac death. HF hospitalization was defined as unscheduled hospital admission requiring HF treatment.

### 2.6 Statistical analysis

Statistical analysis data were presented as mean ± standard deviation or median (interquartile range) for continuous variables and as number and percentage for categorical variables. Continuous and categorical variables were compared using Student's *t*-test or Mann–Whitney *U* test and the χ^2^ test or Fisher's exact test, respectively. Cumulative survival estimates were calculated using the Kaplan–Meier method and compared using the log-rank test. Because no validated cutoff value for peak exercise LA reservoir strain has been established, the median value was used, whereas the continuous value was also used in Cox regression analyses. Univariate and multivariate Cox proportional hazard regression analyses were performed to identify variables associated with events. Given the limited number of primary endpoint events, the multivariable model was restricted to clinically relevant variables to minimize the risk of overfitting and included age, New York Heart Association class, the prevalence of atrial fibrillation, estimated glomerular filtration rate, E/e’ at peak workload, and LA reservoir strain at peak workload. Hazard ratios (HRs) were shown with 95% confidence intervals (CIs). To assess the robustness of the findings, sensitivity analyses were performed by separately adding LV ejection fraction, diabetes mellitus, and natriuretic peptide levels to the primary multivariate model. Because either B-type natriuretic peptide or N-terminal pro-B-type natriuretic peptide levels was available for each patient, each biomarker was logarithmically transformed and standardized separately (z-score), and standardized natriuretic peptide levels was used. To evaluate whether the prognostic association of peak exercise LA reservoir strain differed according to HF phenotype, patients were categorized as HFpEF (LVEF ≥50%) or HFrEF (LVEF <50%). Cox proportional hazards analyses were performed separately in each group, and the interaction between peak exercise LA reservoir strain and HF phenotype was tested. Statistical significance was set at *P* < 0.05. Statistical analysis was performed with statistical software (JMP version 14.0; SAS Institute Inc., Cary, NC, USA).

Inter- and intra-observer differences were analyzed for 20 randomly selected images. LA reservoir strain was evaluated by two blinded observers and by a single observer at two different time points, both at rest and peak workload. Reliability was calculated using Pearson's correlation coefficient. Variability was calculated as the percentage error of each measurement, defined as the absolute difference between the two measurements divided by their mean value and expressed as a percentage.

## 3 Results

### 3.1 Patient characteristics

Clinical characteristics are shown in Table 1. The mean age of the patients was 67 ± 15 years, and 61% of the patients were males. The prevalence rates of hypertension, dyslipidemia, diabetes mellitus and atrial fibrillation were 33%, 33%, 25% and 25%, respectively. Ninety-one (61%) patients had HFrEF, and 59 (39%) had HFpEF.

**Table 1:** Clinical characteristics.

Variables	All (*n* = 150)	Event (+) (*n* = 49)	Event (−) (*n* = 101)	*P*-value
Age, years	67 ± 15	74 ± 11	64 ± 16	< 0.001
Male gender, *n* (%)	92 (61)	27 (55)	65 (64)	0.271
Body surface area, m^2^	1.63 ± 0.22	1.56 ± 0.21	1.66 ± 0.22	0.017
Body mass index, kg/m^2^	23.3 ± 4.1	23.1 ± 4.4	23.7 ± 3.5	0.324
Systolic blood pressure, mmHg	119 ± 22	115 ± 22	120 ± 23	0.237
Heart rate, bpm	69 ± 12	69 ± 12	69 ± 12	0.968
Hypertension, *n* (%)	50 (33)	16 (33)	34 (34)	0.902
Dyslipidemia, *n* (%)	50 (33)	21 (43)	29 (29)	0.084
Diabetes mellitus, *n* (%)	37 (25)	20 (41)	17 (17)	0.001
Atrial fibrillation, *n* (%)	38 (25)	21 (43)	17 (17)	< 0.001
Ischemic heart disease, *n* (%)	25 (17)	9 (18)	16 (16)	0.707
HF with preserved ejection fraction, *n* (%)	59 (39)	25 (51)	34 (34)	0.041
HF with reduced ejection fraction, *n* (%)	91 (61)	24 (49)	67 (66)	
New York Heart Association class I/II/III, *n* (%)	41 (27) / 72 (48) / 37 (25)	3 (6) / 17 (35) / 29 (59)	38 (38) / 55 (54) / 8 (8)	< 0.001
**Laboratory data**				
Hemoglobin, g/dL	13.2 ± 2.1	12.2 ± 2.0	13.6 ± 2.0	< 0.001
Albumin, g/dL	3.8 ± 0.5	3.6 ± 0.6	3.9 ± 0.5	< 0.001
estimated glomerular filtration rate, mL/min/1.73 m^2^	51 ± 24	39 ± 18	57 ± 24	< 0.001
B-type natriuretic peptide, pg/mL	273 (129–568)	522 (102–370)	231 (101–381)	0.481
N-terminal pro-B-type natriuretic peptide, pg/mL	1015 (393–2787)	1165 (381–2421)	823 (403–2527)	0.127
**Medical therapy**				
Angiotensin-converting enzyme inhibitors, angiotensin receptor blockers, or angiotensin receptor neprilysim inhibitors, *n* (%)	106 (71)	31 (63)	75 (74)	0.172
Beta-blockers, *n* (%)	129 (86)	46 (94)	83 (82)	0.053
sodium-glucose transporter 2 inhibitors, *n* (%)	102 (68)	40 (82)	62 (61)	0.013
Aldosterone antagonists, *n* (%)	119 (79)	42 (85)	77 (65)	0.185
Loop diuretics, *n* (%)	86 (57)	36 (73)	50 (50)	0.005
Tolvaptan, *n* (%)	30 (20)	14 (29)	16 (16)	0.068

Data are presented as mean ± standard deviation, median (interquartile range), or number (%) of patients.

HF, heart failure.

During a median follow-up of 1.1 years (interquartile range 0.7–2.4 years), 49 patients had events (3 cardiovascular death and 46 hospitalization for HF). Patients with events were older and had lower body surface area, higher New York Heart Association class, higher prevalences of atrial fibrillation and diabetes mellitus, and lower hemoglobin and albumin levels and estimated glomerular filtration rate than those without events. The use of sodium glucose transporter 2 inhibitors or loop diuretics was more frequent in patients with events than in those without events.

### 3.2 Echocardiographic findings

TTE parameters are shown in Table 2. There was no significant difference in LV ejection fraction or LV end-diastolic volume between the two groups. E, E/e’, LA volume index, TR pressure gradient, and inferior vena cava diameter were higher in patients with events than in those without events. LA reservoir strain was lower in patients with events than in those without events.

**Table 2:** TTE parameters.

Variables	All (*n* = 150)	Event (+) (*n* = 49)	Event (−) (*n* = 101)	*P*-value
LV end-diastolic diameter, mm	55 ± 9	55 ± 8	55 ± 9	0.991
LV end-systolic diameter, mm	43 ± 12	42 ± 12	43 ± 12	0.442
LV mass index, g/m^2^	122 ± 32	120 ± 31	124 ± 35	0.545
LV end-diastolic volume, mL	133 ± 59	130 ± 59	135 ± 60	0.637
LV end-systolic volume, mL	81 ± 55	74 ± 53	84 ± 56	0.291
LV ejection fraction, %	44 ± 16	47 ± 16	43 ± 16	0.116
Stroke volume, mL	51 ± 20	52 ± 19	49 ± 19	0.432
Cardiac output, mL/min	3.4 ± 1.2	3.5 ± 1.0	3.4 ± 1.3	0.507
E, cm/s	93 ± 40	101 ± 41	86 ± 38	0.004
e’, cm/s	5.2 ± 1.9	5.1 ± 1.7	5.3 ± 2.0	0.681
E/e’	18.7 ± 11.3	22.1 ± 9.6	18.5 ± 9.9	0.018
LA volume index, mL/m^2^	62 ± 36	81 ± 39	58 ± 32	< 0.001
LA reservoir strain, %	13.1 ± 5.1	10.1 ± 4.4	14.5 ± 4.9	< 0.001
LA fractional area change, %	23 ± 8	21 ± 7	28 ± 8	< 0.001
LA ejection fraction, %	32 ± 11	31 ± 7	34 ± 11	< 0.001
Right ventricular end-diastolic area, cm^2^	21 ± 5	21 ± 5	22 ± 5	0.221
Right ventricular end-systolic area, cm^2^	13 ± 3	13 ± 4	13 ± 3	0.695
Right ventricular fractional area change, %	41 ± 6	38 ± 6	41 ± 6	0.017
Mitral regurgitation (moderate or severe), *n* (%)	65 (43)	29 (59)	36 (36)	0.003
Tricuspid regurgitation (moderate or severe), *n* (%)	43 (29)	18 (37)	25 (25)	0.044
TR pressure gradient, mmHg	23 ± 10	26 ± 11	21 ± 10	0.020
Inferior vena cava diameter, mm	13 ± 6	16 ± 7	12 ± 5	< 0.001

Data are presented as mean ± standard deviation or number (%) of patients.

E, early diastolic mitral inflow velocity; e’, early diastolic mitral annular velocity; LA, left atrial; LV, left ventricular; TR, tricuspid regurgitation; TTE, transthoracic echocardiography.

### 3.3 Exercise stress echocardiographic findings

Exercise stress echocardiographic parameters are shown in Table 3. Patients with events exhibited lower exercise tolerance with lower systolic blood pressure and heart rate at peak workload than those without events. At peak workload, higher E, lower e’ and higher E/e’ were observed in patients with events compared to those without events. There was no significant difference in TR pressure gradient at peak workload between the two groups. LA reservoir strain at peak workload was lower in patients with events than in those without events.

**Table 3:** Exercise stress echocardiographic parameters.

Variables	All (*n* = 150)	Event (+) (*n* = 49)	Event (−) (*n* = 101)	*P*-value
Peak workload, W	39 ± 21	31 ± 16	43 ± 22	0.002
Systolic blood pressure, mmHg				
rest	118 ± 22	115 ± 22	120 ± 23	0.236
peak	136 ± 28	129 ± 28	140 ± 27	0.022
Heart rate, bpm				
rest	69 ± 12	69 ± 12	69 ± 12	0.961
peak	93 ± 21	87 ± 19	95 ± 22	0.027
Stroke volume, mL				
rest	51 ± 20	52 ± 19	49 ± 19	0.432
peak	53 ± 19	54 ± 21	52 ± 17	0.633
Cardiac output, mL/min				
rest	3.4 ± 1.2	3.5 ± 1.0	3.4 ± 1.3	0.507
peak	4.8 ± 1.9	4.6 ± 1.8	4.9 ± 1.9	0.334
E, cm/s				
rest	93 ± 40	101 ± 41	86 ± 38	0.004
peak	116 ± 44	128 ± 49	109 ± 40	0.012
e’, cm/s				
rest	5.2 ± 1.9	5.1 ± 1.7	5.3 ± 2.0	0.681
peak	5.9 ± 2.2	5.3 ± 1.6	6.3 ± 2.4	0.023
E/e’				
rest	18.7 ± 11.3	22.1 ± 9.6	18.5 ± 9.9	0.018
peak	22.6 ± 11.5	25.2 ± 11.7	19.1 ± 11.3	0.007
LA reservoir strain, %				
rest	13.1 ± 5.1	10.1 ± 4.4	14.5 ± 4.9	< 0.001
peak	17.0 ± 6.4	11.6 ± 4.9	19.8 ± 5.3	< 0.001
LA volume index, mL/m^2^				
rest	66 ± 36	81 ± 39	58 ± 32	< 0.001
peak	66 ± 34	76 ± 36	62 ± 32	< 0.001
TR pressure gradient, mmHg				
rest	23 ± 10	26 ± 11	21 ± 10	0.020
peak	37 ± 16	39 ± 17	35 ± 16	0.148

Data are presented as mean ± standard deviation.

E, early diastolic mitral inflow velocity; e’, early diastolic mitral annular velocity; LA, left atrial; TR, tricuspid regurgitation.

Exercise-induced changes in echocardiographic parameters between rest and peak workload are shown in Table 4. Lower heart rate response was observed in patients with events. The change in E/e’ or TR pressure gradient did not significantly differ between the two groups. The increase in LA reservoir strain was lower in patients with events than in those without events.

**Table 4:** Exercise-induced changes in echocardiographic parameters.

Variables	All (*n* = 150)	Event (+) (*n* = 49)	Event (−) (*n* = 101)	*P*-value
Δ Systolic blood pressure, mmHg	18 ± 21	14 ± 21	20 ± 21	0.081
Δ Heart rate, bpm	24 ± 17	18 ± 16	26 ± 18	< 0.001
Δ Stroke volume, mL	2 ± 1	2 ± 1	3 ± 1	0.782
Δ Cardiac output, mL/min	1.4 ± 1.3	1.1 ± 1.2	1.6 ± 1.2	0.475
Δ E, cm/s	22 ± 24	23 ± 25	22 ± 23	0.823
Δ e’, cm/s	0.6 ± 1.4	0.2 ± 1.2	0.8 ± 1.4	0.009
Δ E/e’	2.4 ± 5.9	3.3 ± 6.2	1.9 ± 5.7	0.178
Δ LA reservoir strain, %	3.8 ± 3.1	1.5 ± 1.6	4.8 ± 3.1	< 0.001
Δ LA volume index, mL/m^2^	0.0 ± 17.2	−5.3 ± 17.8	2.6 ± 16.3	0.011
Δ TR pressure gradient, mmHg	14 ± 13	14 ± 13	15 ± 13	0.791

Data are presented as mean ± standard deviation.

E, early diastolic mitral inflow velocity; e’, early diastolic mitral annular velocity; LA, left atrial; TR, tricuspid regurgitation.

### 3.4 Association of LA reservoir strain with events

**Fig. 3:**
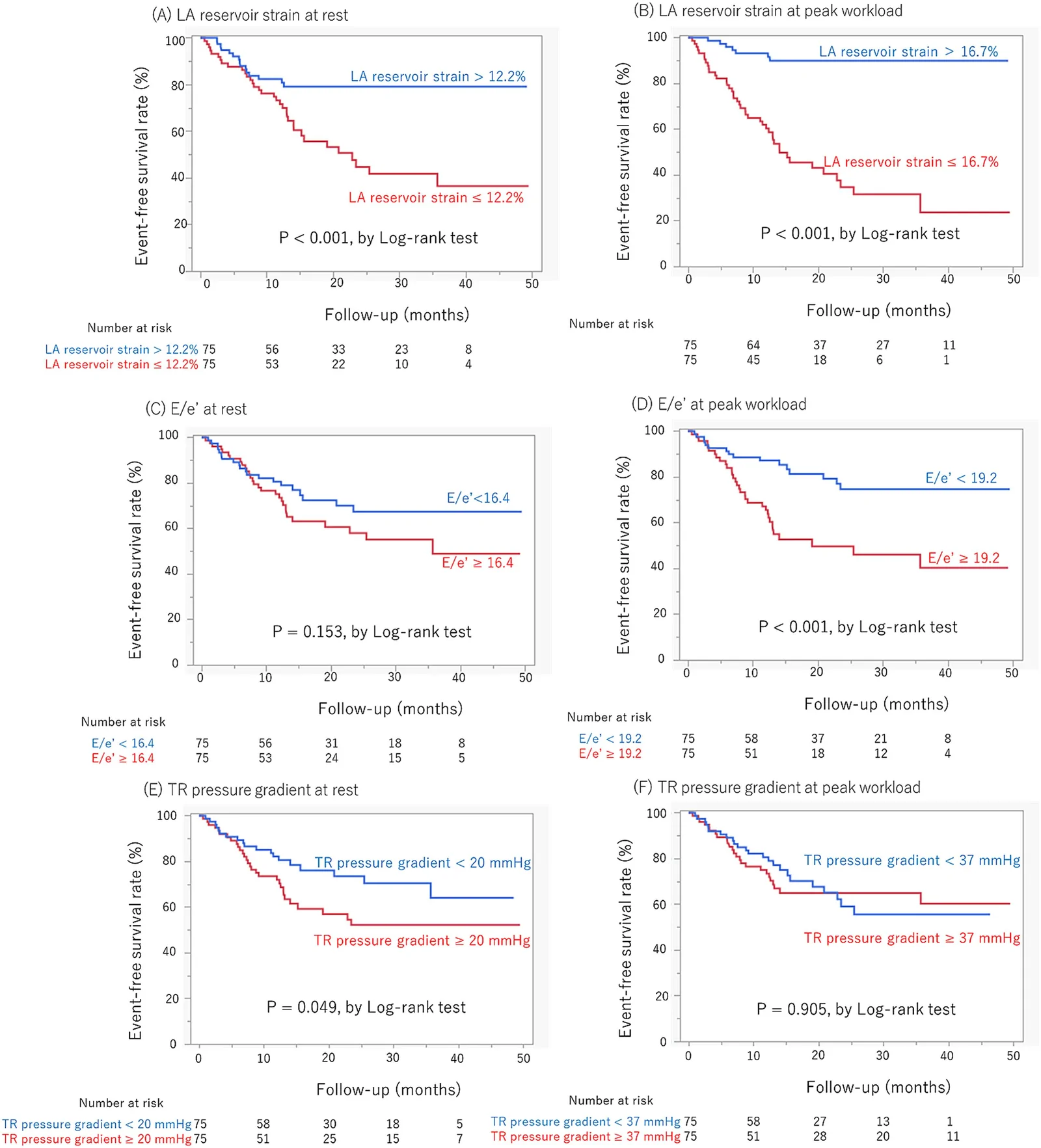
Kaplan–Meier curves for event-free survival. Event-free survival stratified by LA reservoir strain at rest (A), LA reservoir strain at peak workload (B), E/e’ at rest (C), E/e' at peak workload (D), TR pressure gradient at rest (E), and TR pressure gradient at peak workload (F). Log-rank *P*-values are presented in each panel. E, early diastolic transmitral inflow velocity; e’, early diastolic mitral annular velocity; LA, left atrial; TR, tricuspid regurgitation.

Patients were categorized into two groups on the basis of the median values of LA reservoir strain at rest of 12.2% and peak workload of 16.7%: low LA reservoir strain at rest ≤12.2% vs. high LA reservoir strain at rest >12.2%; low LA reservoir strain at peak workload ≤16.7% vs. high LA reservoir strain at peak workload >16.7%. Kaplan–Meier curves showed that patients with low LA reservoir strain at rest had a higher event rate than those with high LA reservoir strain (log-rank test, *P* < 0.001; Fig. 3A). Patients with low LA reservoir strain at peak workload had a higher event rate than those with high LA reservoir strain (log-rank test, *P* < 0.001; Fig. 3B).

Patients were categorized into two groups on the basis of the median values of E/e’ at rest of 16.4 and peak workload of 19.2 or TR pressure gradient at rest of 20 mmHg and peak workload of 37 mmHg. There was no significant difference in the event-free survival rate between patients with high E/e’ at rest and those with low E/e’ (log-rank test, *P* = 0.153; Fig. 3C), while patients with high E/e’ at peak workload had a higher event rate than those with low E/e’ (log-rank test, *P* < 0.001; Fig. 3D). Patients with high TR pressure gradient at rest had a higher event rate than those with low TR pressure gradient (log-rank test, *P* = 0.049: Fig. 3E), but no significant difference in the event-free survival rate was observed between patients with high TR pressure gradient at peak workload and those with low TR pressure gradient (log-rank test, *P* = 0.905; Fig. 3F).

Cox proportional hazard regression analyses were performed to evaluate the determinants of events (Table 5). In univariate Cox regression analysis, age, New York Heart Association class, the prevalence of atrial fibrillation, estimated glomerular filtration rate, LA reservoir strain at rest ≤12.2%, E/e’ at rest ≥16.4, LA reservoir strain at peak workload ≤16.7%, and E/e’ at peak workload ≥19.2 were significant determinants of events. In multivariate analysis, LA reservoir strain at peak workload was independently associated with events (HR: 5.57, 95% CI: 2.74–11.3, *P* < 0.001). LA reservoir strain at peak workload analyzed as a continuous variable was independently associated with events (HR: 2.63 per 5% decrease, 95% CI: 1.93–3.60, *P* < 0.001) (Supplementary S1). In sensitivity analyses, LA reservoir strain at peak workload remained independently associated with events after adjustment for LV ejection fraction (HR: 2.30, 95% CI: 1.67–3.18, *P* < 0.001), diabetes mellitus (HR: 2.25, 95% CI: 1.63–3.11, *P* < 0.001), and standardized natriuretic peptide levels (HR: 2.37, 95% CI: 1.72–3.25, *P* < 0.001) (Supplementary Table S2). LA volume index at rest and peak workload and ΔLA volume index were associated with the primary endpoint in univariable analyses. However, after adjustment for LA reservoir strain at peak workload, none remained independently associated with the primary endpoint (Supplementary Table S3).

**Table 5:** Univariate and multivariate analyses of the predictors of events.

	Univariate HR (95%CI)	*P*-value	Multivariate analysis 1 HR (95%CI)	*P*-value	Multivariate analysis 2 HR (95%CI)	*P*-value
Age, years	1.05 (1.02–1.07)	< 0.001	1.02 (0.99–1.05)	0.307	1.00 (0.98–1.04)	0.512
New York Heart Association class ≥ III	2.82 (1.49–5.33)	< 0.001	1.70 (0.86–3.35)	0.132	1.59 (0.81–3.14)	0.188
Atrial fibrillation	2.64 (1.50–4.66)	< 0.001	1.47 (0.79–2.72)	0.226	1.63 (0.88–3.02)	0.125
Estimated glomerular filtration rate, mL/min/1.73 m^2^	0.97 (0.96–0.99)	< 0.001	1.00 (0.98–1.02)	0.971	1.00 (0.98–1.02)	0.981
LA reservoir strain at rest ≤12.2%	3.60 (1.99–6.62)	< 0.001	1.39 (0.61–3.18)	0.434		
E/e’ at rest ≥16.4	2.97 (1.69–5.24)	< 0.001				
LA reservoir strain at peak workload ≤16.7%	8.33 (4.39–15.8)	< 0.001	8.09 (3.26–20.09)	< 0.001	5.57 (2.74–11.3)	< 0.001
E/e’ at peak workload ≥19.2	3.37 (1.81–6.30)	< 0.001			1.89 (0.96–3.72)	0.065

CI, confidence interval; E, early diastolic mitral inflow velocity; e’, early diastolic mitral annular velocity; HR, hazard ratio; LA, left atrial.

**Fig. 4:**
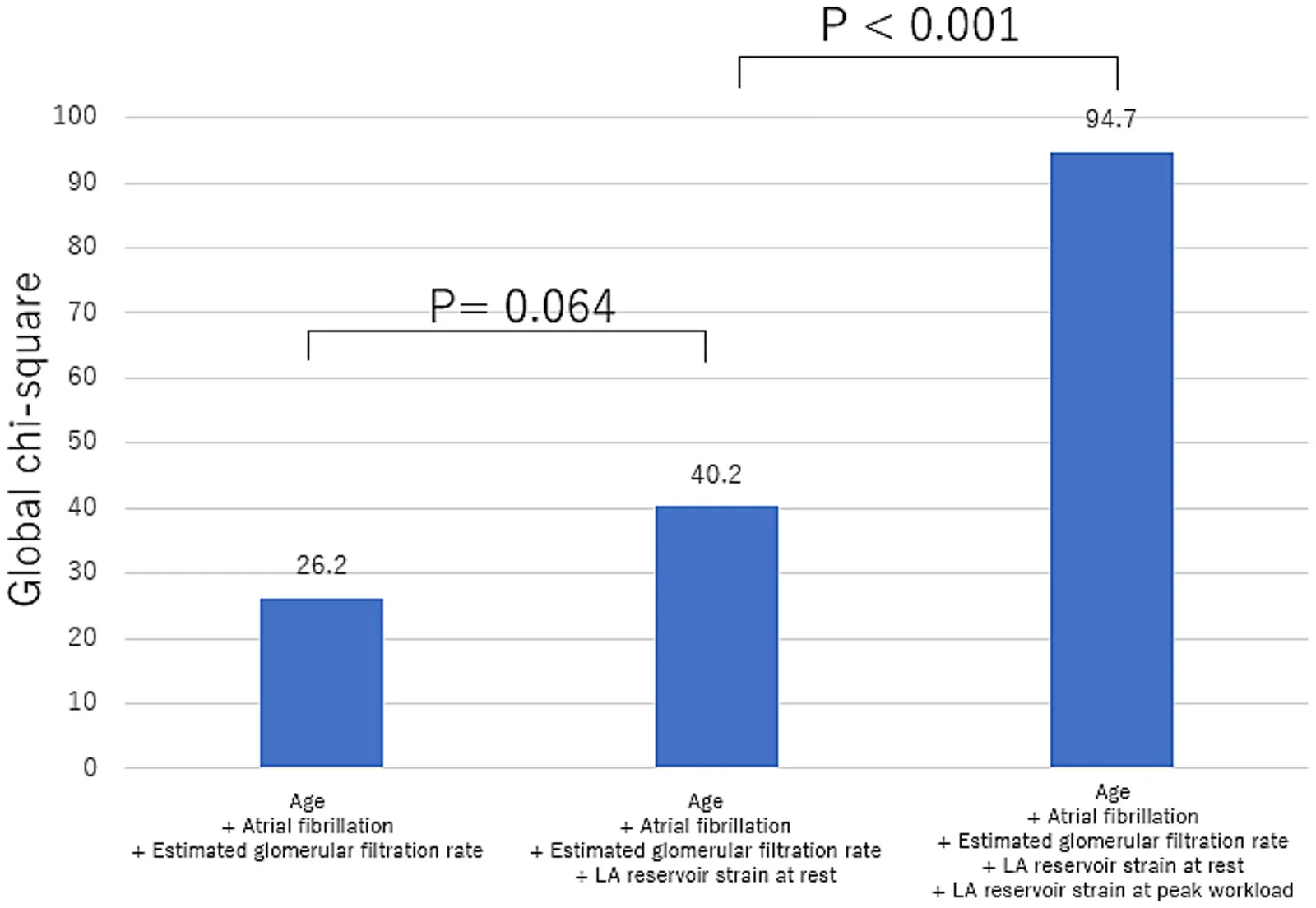
Incremental prognostic value of LA reservoir strain. Global chi-square values derived from Cox proportional hazards are shown for the models, including age, the prevalence of atrial fibrillation and estimated glomerular filtration rate, with the sequential addition of LA reservoir strain at rest and peak workload. LA, left atrial.

The incremental prognostic value of LA reservoir strain at peak workload was evaluated on the basis of clinical factors, including age, atrial fibrillation, and estimated glomerular filtration rate. The addition of LA reservoir strain at peak workload significantly improved the global chi-square value (26.2 to 94.7, *P* < 0.001; Fig. 4). Consistent findings were observed using the C-index (0.669 to 0.770; likelihood ratio test, *P* < 0.001), the integrated discrimination improvement (0.204, 95% CI 0.073–0.342; *P* < 0.001), and continuous net reclassification improvement (0.450, 95% CI 0.155–0.599; *P* = 0.004) (Supplementary Table S4).

### 3.5 LA reservoir strain according to HF phenotypes

**Fig. 5:**
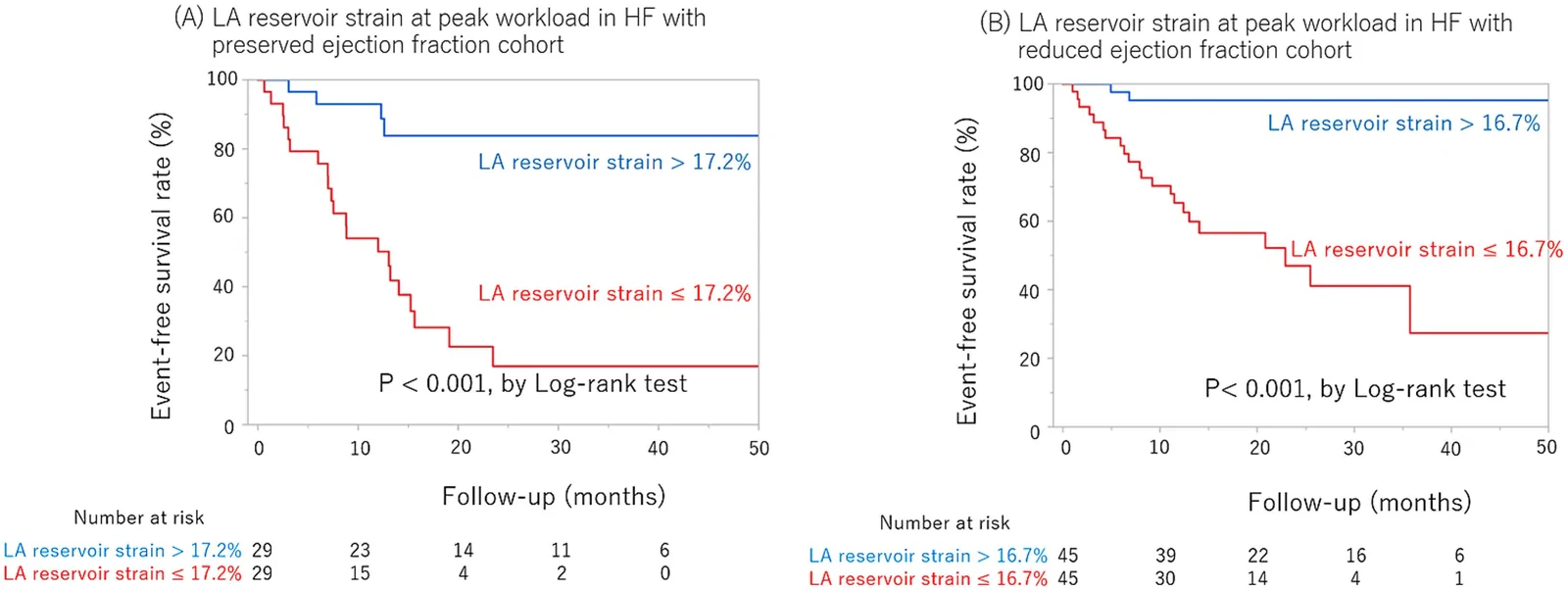
Kaplan–Meier curves for event-free survival according to HF phenotypes. Event-free survival stratified by LA reservoir strain at peak workload in patients with HF with preserved ejection fraction (A) and those with HF with reduced ejection fraction (B). Log-rank *P*-values are presented in each panel. HF, heart failure; LA, left atrial.

According to HF phenotypes, patients were categorized into two groups on the basis of the median values of LA reservoir strain at peak workload of 17.2% in the HFpEF cohort and 16.7% in the HFrEF cohort. Kaplan–Meier curves showed that patients with low LA reservoir strain at peak workload had a higher event rate than those with high LA reservoir strain in both HFpEF and HFrEF cohorts (log-rank test, *P* < 0.001; Fig. 5).

**Supplementary Fig. S1:**
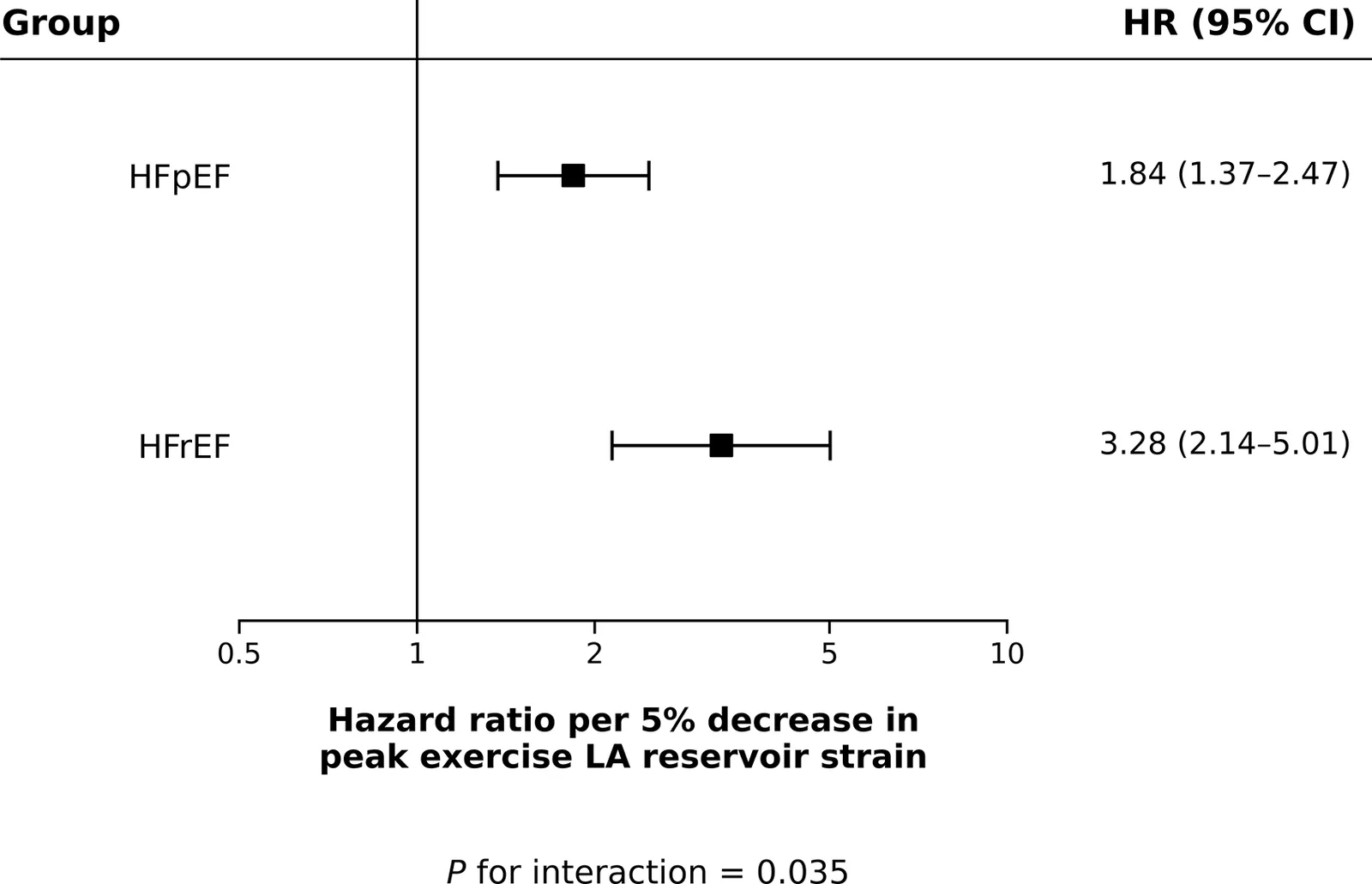
. Association between peak exercise left atrial reservoir strain and the primary endpoint according to HF phenotype. Forest plot showing the association between peak exercise LA reservoir strain (per 5% decrease) and the primary endpoint in patients with HFpEF and HFrEF. A significant interaction between heart failure phenotype and peak exercise LA reservoir strain was observed (P for interaction = 0.035). HF, heart failure; HFpEF, heart failure with preserved ejection fraction; HFrEF, heart failure with reduced ejection fraction; LA, left atrial.

To determine whether the prognostic value of LA reservoir strain at peak workload differed according to HF phenotype, subgroup analyses were performed in patients with HFpEF and those with HFrEF. LA reservoir strain at peak workload was significantly associated with the primary endpoint in both HFpEF (HR: 1.84 per 5% decrease, 95% CI: 1.37–2.47, *P* < 0.001) and HFrEF (HR: 3.28 per 5% decrease, 95% CI: 2.14–5.01, *P* < 0.001). A significant interaction was observed between HF phenotype and LA reservoir strain at peak workload (*P* for interaction = 0.035), indicating a stronger association in patients with HFrEF (Supplementary Fig. S1).

### 3.6 Reproducibility

Inter- and intra-observer reproducibility of LA reservoir strain measurements were assessed in 20 randomly selected images. LA reservoir strain was independently analyzed by two experienced observers who were blinded to the patients' clinical data, and by the same observer at two different time points. LA reservoir strain at rest showed excellent inter-observer agreement (*R* = 0.96, *P* < 0.001) and intra-observer agreement (*R* = 0.94, *P* < 0.001). Similarly, at peak workload, excellent agreement was observed for both inter-observer (*R* = 0.93, *P* < 0.001) and intra-observer measurements (*R* = 0.94, *P* < 0.001). Measurement variability, expressed as percentage error, was low and acceptable for both rest and peak workload measurements, indicating high reproducibility of LA reservoir strain assessment during exercise stress echocardiography.

## 4 Discussion

The present study evaluated the prognostic value of LA function during exercise in patients with HF. The major findings demonstrate that impaired LA reservoir strain at peak workload independently predicted the composite endpoint of cardiovascular death or hospitalization for HF. Importantly, peak exercise LA reservoir strain was significantly associated with the primary endpoint in both HFpEF and HFrEF.

### 4.1 LA function in HF

The LA serves two primary roles. LA function regulates LV filling as a reservoir, conduit, and booster pump, maintaining LV preload and supporting cardiac output, and prevents pulmonary congestion by accommodating venous return from the lungs through its compliant nature, helping to buffer pressure increases. Impaired LA function results in elevated pulmonary capillary pressures and pulmonary arterial hypertension [18].

LA reservoir function is a valuable prognostic indicator in patients with HF. Freed BH et al. reported that reduced LA reservoir strain was associated with increased pulmonary vascular resistance, decreased peak oxygen consumption, and cardiovascular hospitalization or death in patients with HFpEF [19]. Morris et al. reported that peak atrial longitudinal strain was a predictor of mortality and HF hospitalization in patients with HFrEF, independently of LA volume and LV function [20]. These findings indicate the clinical usefulness for assessing LA reservoir function in HF.

### 4.2 LA function during exercise

LA reservoir function increases during exercise, which leads to accelerated LV filling by maintaining an enhanced atrioventricular pressure gradient [21], [22]. Under normal conditions, the LA expands to enhance its reservoir function during exercise, effectively accommodating increased pulmonary venous return without causing a significant increase in LA pressure. However, when LA compliance decreases, this reservoir capacity becomes impaired, resulting in a blunted increase in LA reservoir strain. Consequently, LA pressure increases substantially during exercise, which leads to elevated LV filling pressure [23], [24]. Sugimoto et al. reported that high LA strain during exercise was correlated with high cardiac output in patients with HFpEF, and that a reduction in LA strain during exercise was associated with worse clinical outcomes [10]. Kagami et al. reported that impaired LA reservoir strain during exercise was associated with poor biventricular systolic reserve and cardiac output augmentation, low peak oxygen consumption, and an increased risk of HF events in patients with HFpEF [25]. Although previous studies evaluating exercise LA reservoir function have primarily focused on patients with HFpEF [10], [25], LA dysfunction may also have important prognostic implications in HFrEF despite differences in the mechanisms of atrial remodeling [20]. In HFrEF, impaired LV systolic function, functional mitral regurgitation, and chronically elevated LV filling pressures promote LA enlargement, myocardial fibrosis, and reduced LA compliance [18], [23]. During exercise, increased pulmonary venous return imposes an additional hemodynamic burden on the left atrium, and failure to augment LA reservoir function may therefore reflect impaired atrioventricular compliance, elevated LV filling pressure, and limited cardiac reserve [18], [23]. These pathophysiological mechanisms may explain why peak exercise LA reservoir strain was associated with clinical outcomes in both HFpEF and HFrEF.

### 4.3 Present study

The present study showed that LA reservoir strain at peak workload, rather than at rest, independently predicted adverse outcomes. LA reservoir strain at peak workload had significant incremental prognostic value beyond established clinical factors. Previous studies have demonstrated that resting LA reservoir strain predicts adverse outcomes in both HFpEF and HFrEF [19], [20], whereas investigations of exercise LA reservoir strain have largely been limited to HFpEF [10], [25]. Our phenotype-specific analyses extend these observations by demonstrating that peak exercise LA reservoir strain was significantly associated with events in both HFpEF and HFrEF. These findings suggest that exercise LA reservoir strain reflects a common hemodynamic consequence of HF, integrating LA compliance, LV filling pressure, and cardiovascular reserve beyond resting echocardiographic assessment. Our findings suggest the utility of dynamic LA reservoir function using exercise stress echocardiography as a sensitive prognostic indicator in patients with HF. LA reservoir strain during exercise may improve risk stratification and contribute to therapeutic decision-making in clinical practice. In addition, although LA volume index at rest and peak workload were associated with events in univariable analyses, none remained independently associated after adjustment for LA reservoir strain at peak workload. These findings suggest that dynamic assessment of LA function during exercise provides greater prognostic information than structural LA remodeling alone. Importantly, the prognostic significance of peak exercise LA reservoir strain remained robust after additional adjustment for LV ejection fraction, diabetes mellitus, and natriuretic peptide levels, indicating that peak exercise LA reservoir strain provides prognostic information beyond established clinical, functional, and biochemical risk markers.

In the present study, LA volume index decreased during exercise in patients with events. The precise mechanism remains uncertain. The measurement variability during peak exercise could not be excluded. However, previous studies reported heterogeneous LA volumetric responses, including a decrease in LA volume index, as well as impaired augmentation of LA reservoir function during exercise in patients with HF [10], [26]. The decrease in LA volume index during exercise might reflect the impaired LA volumetric response related to reduced LA compliance.

The present study has clinical strengths. This study enrolled patients with HF, irrespective of LV function, in a real-world clinical setting. The study cohort reflected patients with HF requiring therapeutic interventions, who needed more effective risk assessment regarding the prognosis. This study assessed the effectiveness of LA reservoir strain during exercise in comparison with E/e’ or TR pressure gradient, which were identified as predictors of clinical outcomes in patients with HF. Additionally, exercise was performed using a bicycle ergometer in the supine position, which allowed real-time echocardiographic assessment. Echocardiographic findings, including LA reservoir strain, were accurately evaluated at the time of peak workload, but not after the end of exercise. The prognostic significance of LA reservoir strain at peak workload remained robust when analyzed as a continuous variable, indicating that its predictive value was not dependent on the median-based categorization used for Kaplan–Meier visualization.

### 4.4 Study limitations

The present study had several limitations. First, this was a single center study with a limited sample size. A large study is required to confirm our findings. Second, the follow-up period was short. The number of events was modest. Furthermore, only three cardiovascular deaths occurred during follow-up. Therefore, our study was underpowered to evaluate cardiovascular mortality as an individual endpoint, and the prognostic value of peak exercise LA reservoir strain should be interpreted with respect to the composite endpoint rather than cardiovascular mortality alone. Third, this study involved selection bias because the study population included patients who underwent exercise stress echocardiography. Finally, given the bidirectional relationship between the LA and LV, the influence of LV function on LA reservoir strain could not be assessed.

## 5 Conclusions

LA reservoir strain at peak workload was associated with the composite endpoint of cardiovascular death or hospitalization for HF in both HFpEF and HFrEF and provided incremental prognostic value beyond clinical factors. These findings support the utility of exercise LA reservoir strain for risk stratification in both HFpEF and HFrEF.

The following are the supplementary data related to this article.

Supplementary material 2

Supplementary material 3

Supplementary material 4

Supplementary material 5

## CRediT authorship contribution statement

**Yu Yoshida:** Writing – review & editing, Writing – original draft, Formal analysis, Data curation, Conceptualization. **Yoichi Takaya:** Writing – review & editing, Writing – original draft, Formal analysis, Data curation, Conceptualization. **Rie Nakayama:** Writing – review & editing, Data curation, Conceptualization. **Rika Takemoto:** Writing – review & editing, Data curation. **Eriko Kusunoki:** Writing – review & editing, Data curation. **Norihisa Toh:** Writing – review & editing, Data curation, Conceptualization. **Toru Miyoshi:** Writing – review & editing, Conceptualization. **Shinsuke Yuasa:** Writing – review & editing, Supervision, Conceptualization.

## Author statement

This author takes responsibility for all aspects of reliability and freedom from bias of the data presented and their discussed interpretation.

## Grant support

None.

## Declaration of competing interest

The authors declare that they have no known competing financial interests or personal relationships that could have appeared to influence the work reported in this paper.
