# Supplementary material for: Prognostic significance of left atrial reservoir strain during exercise in patients with heart failure
Source: Int J Cardiol Heart Vasc. 2026 Aug 24;66:102001. doi: 10.1016/j.ijcha.2026.102001 (PMC13542481; doi:10.1016/j.ijcha.2026.102001)
Supplement: Supplementary file 1 [file mmc2.docx]

| Supplementary Table S1 Cox regression analysis of peak exercise LA reservoir strain for events | | |
| --- | --- | --- |
|  | Adjusted HR (95% CI) | *P*-value |
| Peak exercise LA reservoir strain (per 5% decrease) | 2.63 (1.93–3.60) | < 0.001 |
| CI, confidence interval; HR, hazard ratio; LA, left atrial. The multivariate model was adjusted for age, the prevalence of atrial fibrillation, estimated glomerular filtration rate, and peak exercise E/e′. | | |
