# Supplementary material for: Prognostic significance of left atrial reservoir strain during exercise in patients with heart failure
Source: Int J Cardiol Heart Vasc. 2026 Aug 24;66:102001. doi: 10.1016/j.ijcha.2026.102001 (PMC13542481; doi:10.1016/j.ijcha.2026.102001)
Supplement: Supplementary file 2 [file mmc3.docx]

| Supplementary Table S2 Sensitivity analyses of peak exercise LA reservoir strain | | |
| --- | --- | --- |
|  | Adjusted HR (95% CI) | *P*-value |
| Primary multivariate model | 2.63 (1.93–3.60) | < 0.001 |
| Left ventricular ejection fraction | 2.30 (1.67–3.18) | < 0.001 |
| Diabetes mellitus | 2.25 (1.63–3.11) | < 0.001 |
| Standardized natriuretic peptide | 2.37 (1.72–3.25) | < 0.001 |

The primary multivariate model was adjusted for age, the prevalence of atrial fibrillation, estimated glomerular filtration rate, and peak exercise E/e′.

CI, confidence interval; HR, hazard ratio; LA, left atrial.
