# Supplementary material for: Prognostic significance of left atrial reservoir strain during exercise in patients with heart failure
Source: Int J Cardiol Heart Vasc. 2026 Aug 24;66:102001. doi: 10.1016/j.ijcha.2026.102001 (PMC13542481; doi:10.1016/j.ijcha.2026.102001)
Supplement: Supplementary file 3 [file mmc4.docx]

| Supplementary Table S3 Prognostic significance of LA volume index | | | | |
| --- | --- | --- | --- | --- |
|  | Univariable HR (95% CI) | *P*-value | Adjusted HR (95% CI) | *P*-value |
| Rest LA volume index (per 10 mL/m²) | 1.10 (1.04–1.17) | 0.001 | 0.97 (0.91–1.04) | 0.416 |
| Peak LA volume index (per 10 mL/m²) | 1.08 (1.01–1.16) | 0.030 | 0.94 (0.87–1.03) | 0.175 |
| ΔLA volume index  (per 10 mL/m²) | 0.81 (0.69–0.95) | 0.012 | 0.92 (0.78–1.08) | 0.312 |

Adjusted for peak exercise LA reservoir strain.

CI, confidence interval; HR, hazard ratio; LA, left atrial.
