# Supplementary material for: Prognostic significance of left atrial reservoir strain during exercise in patients with heart failure
Source: Int J Cardiol Heart Vasc. 2026 Aug 24;66:102001. doi: 10.1016/j.ijcha.2026.102001 (PMC13542481; doi:10.1016/j.ijcha.2026.102001)
Supplement: Supplementary file 4 [file mmc5.docx]

| Supplementary Table S4 Incremental prognostic value of peak exercise LA reservoir strain by discrimination analyses | | | | | | |
| --- | --- | --- | --- | --- | --- | --- |
| Model comparison | Concordance index | Likelihood ratio test | Integrated discrimination improvement (95% CI) | *P*-value | Continuous net reclassification improvement (95% CI) | *P*-value |
| Clinical model | 0.669 | Reference | Reference | – | Reference | – |
| Clinical model  + rest LA reservoir strain | 0.670 | 0.680 | 0.027  (−0.011 to 0.108) | 0.244 | 0.035  (−0.198 to 0.251) | 0.789 |
| Clinical model  + peak exercise LA reservoir strain | 0.770 | < 0.001 | 0.107  (0.022 to 0.213) | 0.008 | 0.225  (−0.042 to 0.424) | 0.118 |
| Clinical model  + rest LA reservoir strain + peak exercise LA reservoir strain† | – | – | 0.204  (0.073 to 0.342) | < 0.001 | 0.450  (0.155 to 0.599) | 0.004 |

CI, confidence interval; LA, left atrial.
